# Incessant device-initiated wide complex tachycardia: What is the mechanism?

**DOI:** 10.1016/j.hrcr.2024.09.017

**Published:** 2024-09-26

**Authors:** Ilya Y. Shadrin, Kevin L. Thomas, Tristram D. Bahnson

**Affiliations:** 1 Department of Medicine, Division of Cardiology, Duke University Hospital, Durham, NC; 2 Duke Clinical Research Institute, Durham, NC

**Keywords:** AV nodal re-entry tachycardia, pacemaker, arrhythmia, EP study, wide complex tachycardia, atypical AVNRT, post-pacing interval, entrainment

**Key teaching points**

• Unlike typical AVNRT that is frequently induced with premature atrial contractions, atypical AVNRT is commonly initiated with PVCs or ventricular stimulation in the presence of a pacemaker.
• A VA time >70 ms in the presence of an AHA response during atrial entrainment, and a VAV response with a postpacing interval minus tachycardia cycle length >115 ms after ventricular entrainment are diagnostic of atypical AVNRT.
• Variable AA intervals can be seen during atypical AVNRT in the presence of multiple retrograde slow pathways.

## Introduction

Wide complex tachycardia comprises a broad group of tachyarrhythmias in patients with cardiac implantable electronic devices. Standard maneuvers for establishing the correct diagnosis require careful interpretation in the presence of ventricular pacing. Furthermore, atypical forms of common arrhythmias may require a combination of maneuvers to be performed during an electrophysiology study to correctly identify the culprit arrhythmia and localize the appropriate region for targeted ablation. In this report, we describe a patient with a biventricular defibrillator who presented with incessant wide-complex tachycardia with associated ventricular pacing.

## Case presentation

**Figure 1:**
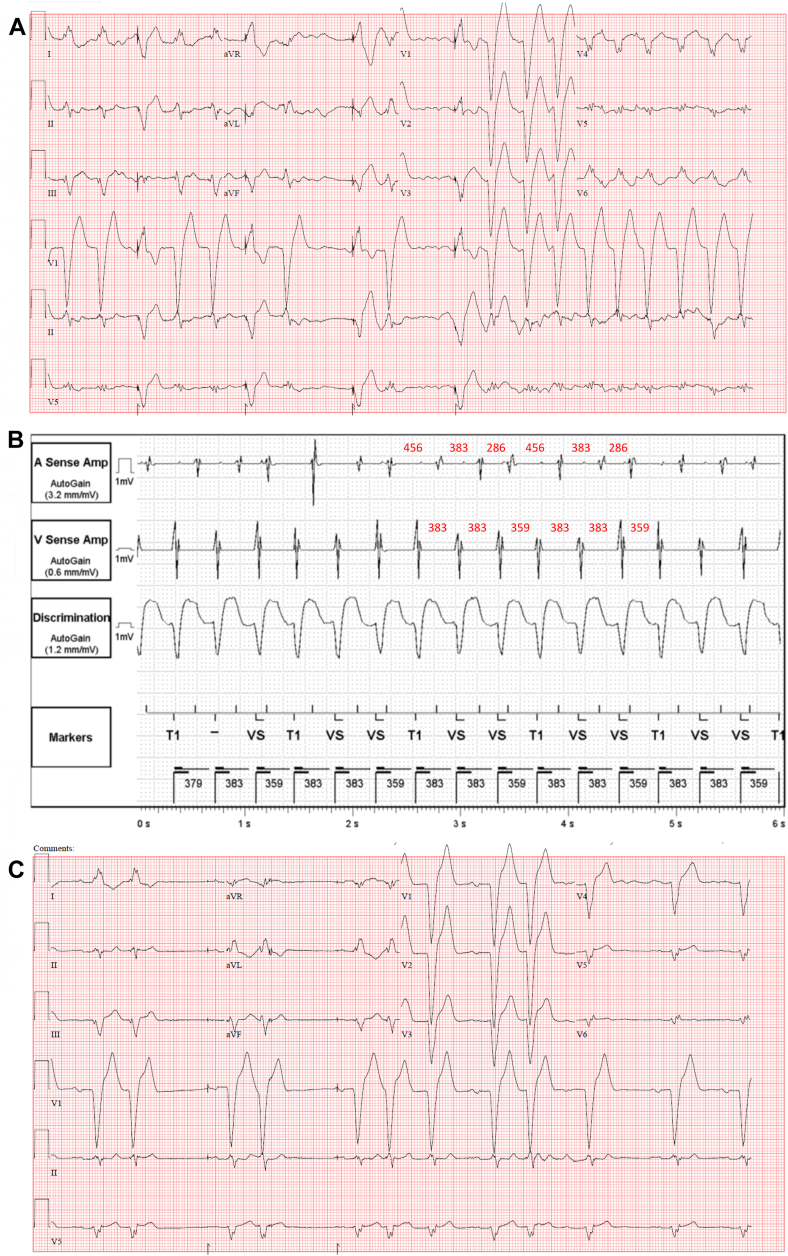
**A:** Standard 12-lead ECG obtained at baseline with wide complex single premature beats, couplets, and onset of wide complex tachycardia. All initial wide complex beats are preceded by a paced beat. **B:** Device electrograms during tachycardia episode show fluctuating VV and AA intervals with a possible repeating pattern. **C:** 12-Lead ECG with V-pacing inhibited on device showing the morphology of intrinsic ventricular activation. Note that the instances of wide complex couplets have a uniform morphology identical to intrinsic ventricular activation.

**Figure 2:**
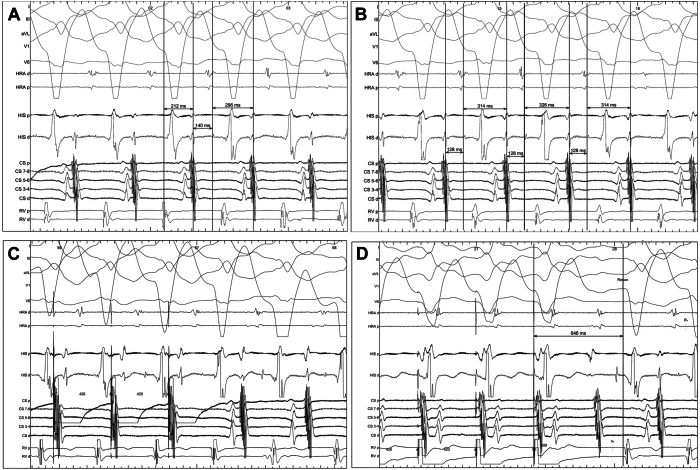
**A, B:** Sustained tachycardia showing intervals at various sweep speeds (100 mm/s in **A,** 67 mm/s in **B**). **C:** Atrial overdrive pacing from the proximal CS. **D:** Entrainment during tachycardia from the RV apex.

A 74-year-old man with a history of heart failure with recovered ejection fraction, left bundle branch block (LBBB), and a cardiac resynchronization therapy with a defibrillator (Abbott, Abbott Park, IL) presented to the emergency department with numerous episodes of presyncope and an irregular rhythm on examination. Transthoracic echocardiography showed new moderate reduction in left ventricular ejection fraction to 40%. Twelve-lead electrocardiogram (ECG) demonstrated intermittent biventricular pacing, with some paced beats followed by salvos of a regular wide complex tachycardia lasting 2–10 seconds at a time (Figure 1A), which reproduced his symptoms. His underlying rhythm was sinus with LBBB. Device interrogation revealed numerous episodes of tachycardia with variable ventricular cycle lengths (CL) of 359–383 ms and irregular A-A intervals of 286–456 ms (Figure 1B). Tachycardia was not perturbed by intravenous lidocaine (100-mg bolus) but was transiently suppressed with intravenous adenosine (12-mg bolus). Couplets and triplets of similar morphology to tachycardia were also noted on 12-lead ECG during inhibition of biventricular pacing (Figure 1C). The patient was taken for an electrophysiologic study, which demonstrated an AH jump during atrial extrastimulation (sinus rhythm, AH 154 ms). Wide complex tachycardia (CL 446 ms) was easily induced with atrial extrastimulation (S1S2 600/435 ms) from the proximal coronary sinus (CS). One-to-one VA conduction, a VA interval of 212 ms, and concentric CS atrial activation were noted during tachycardia (Figure 2A), with variability in atrial CLs despite stable AH intervals of 128 ms (Figure 2B). Atrial overdrive pacing from the proximal CS and entrainment from the right ventricular apex are shown in Figure 2C and 2D. What is the mechanism of the patient’s clinical tachycardia?

## Discussion

The differential diagnosis for regular wide complex tachycardia includes ventricular tachycardia (VT), sinus tachycardia or supraventricular tachycardia (SVT) with aberrancy or ventricular pre-excitation, and a paced rhythm. Device interrogation identified absence of V-pacing during tachycardia, and salvos of tachycardia made sinus tachycardia unlikely. Although the discordance between relatively regular VV intervals and irregular AA intervals suggests possible VT with periods of 1:1 VA conduction and intermittent VA delay, the QRS morphology of tachycardia was identical to that during normal sinus rhythm with the pacemaker inhibited (LBBB pattern), and the clinical tachycardia terminated with adenosine but not lidocaine, arguing against VT. After adenosine-mediated tachycardia termination, ventricular pacing was observed without induction of arrhythmia, again implicating the AV node in the tachycardia mechanism. In this case, SVT with LBBB aberrancy could be a focal atrial tachycardia (AT), atrioventricular nodal re-entry tachycardia (AVNRT), or junctional tachycardia, although most ATs are not adenosine sensitive. Atrial entrainment with overdrive pacing from the proximal CS elicited an AHA response rather than an HA response (Figure 2C), ruling out junctional tachycardia. Ventricular entrainment (Figure 2D) indicated a VAV response of the last entrained A with a long VA interval, eliminating AT because of absence of a VAAV response. The long VA time of 212 ms (>70 ms) could be consistent with either atypical AVNRT or orthodromic atrioventricular reciprocating tachycardia (AVRT), because antidromic AVRT would exhibit a pre-excitation pattern different from typical LBBB. No His-refractory premature ventricular complexes (PVCs) terminated the arrhythmia, which would have supported an alternate diagnosis of AVRT. Instead, after ventricular entrainment, the time difference between the postpacing interval and TCL was 200 ms; given this exceeded an established cutoff of 115 ms,^1^ a diagnosis of atypical (fast-slow) AVNRT with variable VA times was made, because the patient had an AH/HA ratio <1 (140 ms/296 ms, Figure 2A), with an AH interval similar during AVNRT and sinus rhythm (140 ms vs 154 ms, respectively). The patient underwent successful ablation in the region of the slow pathway, which eliminated echo beats and tachycardia inducibility, and prevented clinical recurrence of his tachycardia, confirming the diagnosis.

The diagnosis of AVNRT was made using a combination of bedside pharmacologic and laboratory-based pacing maneuvers. Notably, the tachycardia was reproducibly terminated (albeit temporarily) with adenosine. The pronounced irregularity in the atrial TCL seen on both device interrogation (Figure 1B) and intracardiac EGMs (Figure 2B) in the context of AVNRT is in contrast to Wenckebach physiology because of the pattern of progressively shortening (rather than lengthening) AA intervals every 3 beats. The stability of the AH interval (128 ms, Figure 2B) in the presence of varying HA intervals suggests varying degrees of conductivity (eg, multiple slow pathways) across the retrograde limb of the tachycardia circuit. In contrast, less HV variability is noted across successive beats, although underlying infrahisian disease causing intermittent delay could not be ruled out. Clinically, although typical AVNRT is most often triggered by a premature atrial contraction, atypical AVNRT is more easily triggered by early ventricular stimulation (PVC or V-pacing). In this case, the patient had frequent AVNRT that followed a biventricular paced beat, with considerably less occurrence when V-pacing was inhibited on the device. This suggested that the retrograde VA conduction with V-pacing was timed appropriately to enter the excitable gap between the slow and fast pathways and initiate the tachycardia with findings at intracardiac electrophysiology study consistent with atypical AVNRT.

## Disclosures

IS has previously received grants from the NIH/NHLBI and AHA. TB reports grants from the NIH/NHLBI, St. Jude Medical Inc, Abbott Medical, Biosense Webster Inc, Johnson & Johnson, and Boston Scientific Corp, and consulting from Cardiofocus Inc. and Ventrix. KT is on advisory boards of Boston Scientific Corp, BMS, Janssen, and Biosense Webster Inc.
